# Rapamycin Inhibition of Polyposis and Progression to Dysplasia in a Mouse Model

**DOI:** 10.1371/journal.pone.0096023

**Published:** 2014-04-24

**Authors:** Karin M. Hardiman, Jianhua Liu, Ying Feng, Joel K. Greenson, Eric R. Fearon

**Affiliations:** 1 Department of Surgery, University of Michigan Medical School, Ann Arbor, Michigan, United States of America; 2 Department of Internal Medicine, University of Michigan Medical School, Ann Arbor, Michigan, United States of America; 3 Department of Human Genetics, University of Michigan Medical School, Ann Arbor, Michigan, United States of America; 4 Department of Pathology, University of Michigan Medical School, Ann Arbor, Michigan, United States of America; University of Munich, Germany

## Abstract

Familial adenomatous polyposis (FAP) is often due to *adenomatous polyposis coli* (*APC*) gene germline mutations. Somatic *APC* defects are found in about 80% of colorectal cancers (CRCs) and adenomas. Rapamycin inhibits mammalian target of rapamycin (mTOR) protein, which is often expressed in human adenomas and CRCs. We sought to assess the effects of rapamycin in a mouse polyposis model in which both *Apc* alleles were conditionally inactivated in colon epithelium. Two days after inactivating *Apc*, mice were given rapamycin or vehicle in cycles of two weeks on and two weeks off. Polyps were scored endoscopically. Mice were euthanized at time points or when moribund, and tissue analyses were performed. In other studies, mice with demonstrable *Apc*-defective colon polyps were given rapamycin, followed by analysis of their colon tissues. The median survival of mice receiving rapamycin treatment cycles was 21.5 versus 6.5 weeks in control mice (p = 0.03), and rapamycin-treated mice had a significantly lower percentage of their colon covered with polyps (4.3+/− 2 vs 56.5+/− 10.8 percent, p = 0.001). Mice with *Apc*-deficient colon tissues that developed high grade dysplasia treated with rapamycin underwent treatment for significantly longer than mice treated with vehicle (15.8 vs 5.1 weeks, p = 0.003). In *Apc*-defective colon tissues, rapamycin treatment was linked to decreased levels of β-catenin and Sox9 at 7 weeks. Other effects of rapamycin in *Apc*-defectivecolon tissues included decreased proliferation and increased numbers of differentiated goblet cells at 7 weeks. Rapamycin did not affect β-catenin-regulated gene expression in cultured intestinal epithelial cells. Rapamycin has potent inhibitory effects in a mouse colon polyposis model, and mTOR inhibition is linked to decreased proliferation and increased expression of differentiation markers in *Apc*-mutant colon epithelium and delays development of dysplasia. Our findings highlight the possibility that mTOR inhibitors may have relevance for polyposis inhibition approaches in FAP patients.

## Introduction

One of the major clinical challenges in most patients with familial adenomatous polyposis (FAP) is the development of thousands of colorectal adenomas which can progress to carcinoma without proctocolectomy. Current medical management to prevent colon cancer is not sufficiently efficacious to eliminate the need for prophylactic surgery in FAP patients. Germline mutation of one allele of the *adenomatous polyposis coli* (*APC*) gene underlies the vast majority of FAP cases, with adenomas arising in large part from the somatic inactivation of the remaining wild type copy of the *APC* gene. *APC* inactivation is also seen in 80% of sporadic colorectal adenomas and colorectal cancers (CRCs). The primary means by which *APC* inactivation contributes to colon adenoma and CRC development is believed to be the resultant dysregulation in the levels and localization of the β-catenin protein, particularly the role of nuclear β-catenin as a binding partner for the T cell factor (TCF) family of transcription factors and the function of β-catenin/TCF complexes as key regulators of the expression of certain genes [1].

Rapamycin (RAP) is an inhibitor of the mammalian target of rapamycin protein (mTOR) which is strongly expressed in mouse small intestine polyps with *Apc* defects [2] and in human colorectal adenomas and CRCs [3]. Additionally, a downstream phosphorylation target and likely physiological effector of mTOR signaling, the S6 ribosomal protein, shows increased phosphorylation (phospho-S6) in roughly 40 percent of colorectal carcinomas (CRCs) and adenomas [4]. mTOR regulates cell growth, proliferation, and motility and inhibition inhibits cancer development or growth in various models [5], [6].

The purpose of this study was to assess the ability of RAP to inhibit colon polyps in an *Apc* mutation-dependent colon polyposis model where both copies of the *Apc* gene were somatically inactivated in mouse colon epithelium. We found RAP treatment inhibited colon polyp formation, increased time to progression to dysplasia, and also increased survival. The tissue effects of RAP on *Apc* mutation-induced colon polyps included decreased proliferation and increased differentiation. Future studies may further bolster the case for use of mTOR inhibitors for polyposis inhibition in FAP patients.

## Materials and Methods

### Antibodies and Reagents

Primary antibodies used in this study included: mouse anti-BrdU and mouse anti-β-catenin, BD Biosciences (San Jose, CA); rabbit anti-phospho-S6 ribosomal protein (Ser240/244) XP mAb, rabbit anti-phospho-mTOR (Ser2448) mAb, Cell Signaling (Danvers, MA); mouse anti- β-actin, Sigma-Aldrich (St. Louis, MO); rabbit anti-Sox9, Millipore (Bilerica, MA) and mouse anti-active β-catenin Milipore (Bilerica, MA). The secondary antibodies used were HRP-conjugated anti-rabbit IgG, Cell Signaling and HRP-conjugated anti-mouse IgG, Thermo Scientific (Cincinnati, OH). Dulbecco's Modifid Eagle Medium (DMEM), M199 and cosmic calf serum were from HyClone (Logan, UT). Tamoxifen, Alcian blue, human apo-transferin, hydrocortisone and sodium selenite were obtained from Sigma-Aldrich. Human epithelial growth factor was from Peprotech (Rocky Hill, NJ). Human insulin and 0.05% Trypsin-EDTA were from Invitrogen (Carlsbad, CA). Rapamycin (RAP) was purchased from LC Laboratories (Woburn, MA).

### Cell Line and Culture

The human colonic epithelial cell line (HCEC) was a kind gift from Dr. Jerry W. Shay [7]. HCEC cell lines stably transformed with shAPC (HCEC/ptripzshAPC) or shScramble (HCEC/ptripzshSCR) were established. HCEC cells were seeded in DMEM + Medium 199 (4∶1) supplemented with 2% cosmic calf serum, 20 ng/ml human EGF, 10 ug/ml human recombinant insulin, 2 µg/ml human apo-transferrin, 1 µg/ml hydrocortisone and 5 nM sodium selenite. The cells were treated with Doxycycline (1 µg/ml) or vehicle for 48 hr, then, the cells were treated with RAP (0.001, 0.01, 0.1 and 1 µM) or dimethylsulfoxide vehicle for 24 hours in DMEM + Medium 199 (4∶1) with 0.2% cosmic calf serum, with or without 1 µg/ml of Doxycycline.

### Mice

#### Ethics

We cared for mice and performed experimental procedures in strict accordance with the recommendations in the Guide for the Care and Use of Laboratory Animals of the National Institutes of Health. The protocol was approved by the University Committee on Use and Care of Animals at the University of Michigan and according to Michigan state and US federal regulations. All procedures were performed using isoflurane for anesthesia and all efforts were made to minimize suffering.

#### Model

*CDX2P-CreER^T2^* transgenic mice, expressing a tamoxifen-regulated Cre transgene under control of a roughly 9.5 kb human *CDX2* upstream regulatory element were used. The generation of the *CDX2P-CreER^T2^* transgenic mouse line #741 used here has been described previously [8], though as compared to the *CDX2P-CreER^T2^* transgenic line #752 that was the focus of the paper of Feng and colleagues (9), transgenic line #741 shows increased tamoxifen-regulated Cre function in distal colon epithelium. The *CDX2P-CreER^T2^* mice were intercrossed with mice homozygous for an *Apc* allele containing loxP sites flanking Apc exon 14 (*Apc^fl/fl^*, 580S) [9]. All the mice were housed in specific-pathogen free conditions. After weaning, rodent 5001 chow and automatically supplied water were provided *ad libitum* to mice. At 8–10 weeks of age, *CDX2P-CreER^T2^ Apc^fl/fl^* mice were injected with single dose of tamoxifen (TAM) [0.07 g/kg, intraperitoneal (i.p.)] to activate Cre recombinase. Following Cre-mediated targeting of the *loxP*-containing *Apc* gene, the targeted allele cannot express a functional Apc protein. Injection with RAP (3 mg/kg/day, i.p.) or vehicle was then started 2 days later. Administration of RAP or vehicle was continued daily for two weeks and then stopped for two weeks, and then cycled on and off in two-week intervals. Mice were weighed twice weekly. Mice were euthanized when near moribund or at various time points for tissue studies. In a second group of mice, dense polyps were induced (determined by endoscopy) and the mice were given RAP for 0, 1, 3, or 5 days and euthanized.

### Endoscopy and assessment of polyps

The colon was prepped by water enema introduced trans-anally via transfer pipette. The mice were anesthetized with 1.5% isoflurane. A small animal endoscope (Karl Storz Veterinary Endoscopy, Goleta, CA) was inserted trans-anally and advanced to the bend just proximal to the cecum and videos were recorded. Videos were later viewed and scored in a blinded fashion. Severity of the polyposis was scored according to the following polyp scoring system: 0, no lesion; 1, one small polyp; 2, few small distal polyps; 3, many small distal polyps; 4, many large distal polyps; 5, dense distal polyps. In mice with dense polyposis, the length of the dense area of polyps was measured in centimeters in the explanted colon after euthanasia and fixation of the colon under a dissecting microscope and compared to the total length of the colon excluding the cecum. Hematoxylin and eosin stained sections of the distal colon were assessed by a gastrointestinal pathologist (Joel Greenson, MD) to look for dysplasia.

### Immunohistochemistry

The mice were anesthetized with isoflurane and euthanized at defined time points or when moribund. Colon tissues were then removed and fixed in 10% Formalin overnight, prior to embedding in paraffin. One hr prior to euthanasia, BrdU (0.04 g/kg) was injected i.p. into the mice. Sections of formalin-fixed, paraffin-embedded mouse colon tissues were utilized for immunohistochemical analysis using the following primary antibodies: mouse anti-BrdU (1∶300), mouse anti-β-catenin (1∶800), rabbit anti-phospho-S6 (1∶100), rabbit anti-phospho-mTOR (1∶100) and rabbit anti-Sox9 (1∶1000). Antigen retrieval was performed by heating the sections in microwave in Antigen Retrieval Citra Solution (Biogenex, Fremont, CA) for 15 minutes prior to application of the primary antibodies. Appropriate secondary antibodies were used and the reaction was visualized using diaminobenzidine tetrahydrochloride (DAB) (Vector Laboratories), and cell nuclei were visualized by counterstaining with hematoxylin. BrdU staining was quantified by counting the number of positive and negative cells in at least 20 crypts and the percentage of positive cells per crypt was then calculated. The crypts were then divided into 3 equal parts (top, middle, and bottom) and the quantification was performed again.

### Alcian blue staining

Alcian blue was dissolved in 3% acetic acid to make 1% of Alcian blue solution. Sections were stained in Alcian blue solution for 20 minutes, and counterstained with hematoxylin. Alcian blue staining was quantified by counting the number of positive and negative cells in at least 20 crypts and the percentage of positive cells per crypt was then calculated.

### Western Blot

After treatment with RAP, cells were washed with cold PBS and lysed in RIPA buffer supplemented with complete Mini Protease Inhibitor Cocktail and phosphatase inhibitor cocktail, Roche Applied Science (Indianapolis, IN). Western blotting was performed as previously described [10]. The primary antibodies against active β-catenin [11], phospho-S6, phospho-mTOR and β-actin were used at 1∶1,000 dilutions. Phospho-S6 or β-actin protein expression bands were detected with SuperSignal, West Pico chemiluminescent substrate (Thermo Scientific, Cincinnati, OH) and phospho-mTOR protein expression bands were detected with SuperSignal, West Femto Maximum sensitivity substrate (Thermo Scientific, Cincinnati, OH).

### Quantitative Reverse-Transcription Polymerase Chain Reaction

Total RNA from RAP treated HCEC's was extracted using the RNeasy Mini Kit (Qiagen). The following PCR primers were used: AXIN2, forward, 5′-TTCCCGAGAACCCACCGCCT-3′, reverse, 5′-GCCCCCTCCCGCGAATTGAG-3′; NKD1, forward 5′- CATGCACGACATGAAAATCC-3′, reverse 5′-TTGCCTGGATCTTGGAAAAC-3′; IRS1, forward 5′-CAAGACCATCAGCTTCGTGA-3′, reverse, 5′-AGAGTCATCCACCTGCATCC-3′. Real-time PCR was performed using the QuantiTect SYBR Green RT-PCR kit (Qiagen).

Reverse transcription of mRNA and real-time PCR were performed using the one step QuantiTect SYBR Green RT-PCR kit (Qiagen) according to the manufacturer's protocol. Relative expression of *AXIN2*, *NKD1* and *IRS1* were assessed and normalized with housekeeping gene U6.

### Statistical Analysis

Kaplan-Meier survival curves were generated and analyzed with log rank tests. For the cell culture experiment, two-way ANOVA was used. All other comparisons were performed using t-test or chi-square including 2 sample Z test where appropriate. P values of <0.05 were considered significant. Statistical tests were performed using GraphPad Prism6 software (La Jolla, CA).

## Results

### Rapamycin Improves Survival in an *Apc* Mutation-dependent Mouse Colon Polyposis Model

**Figure 1:**
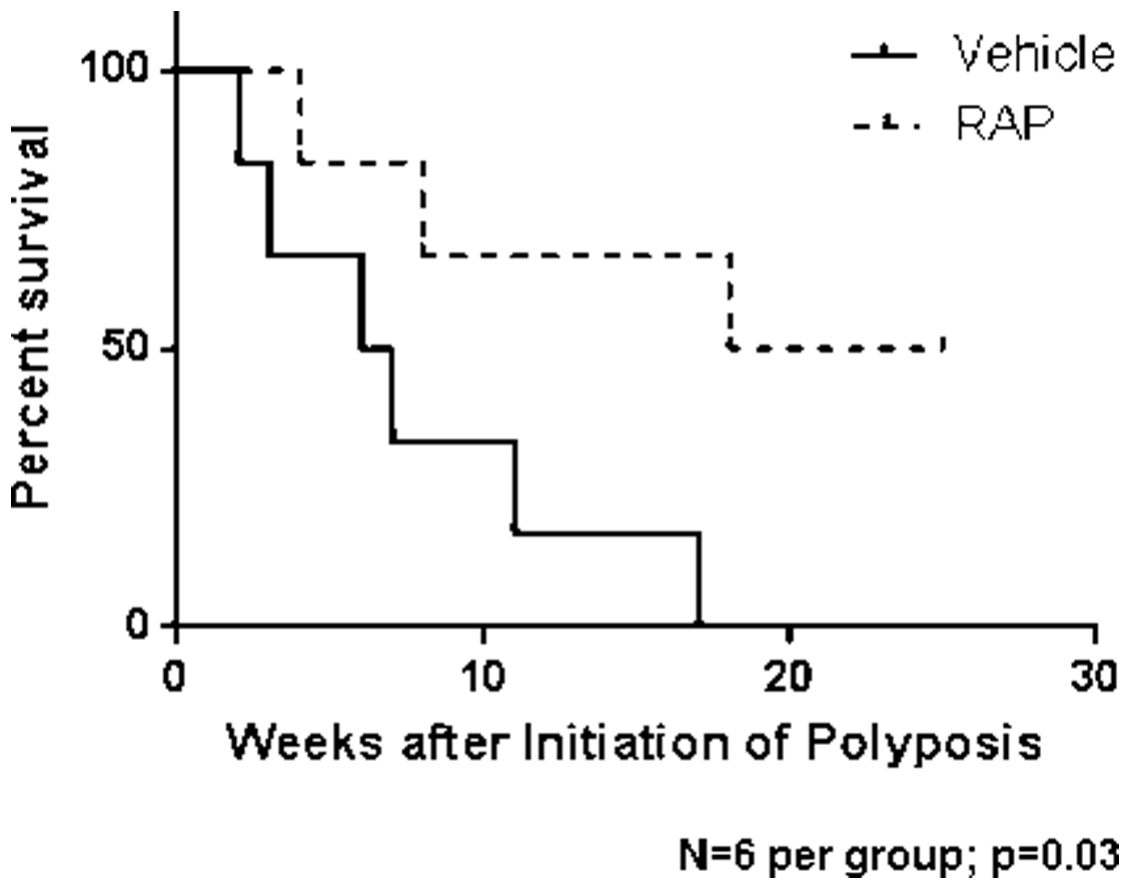
Rapamycin (RAP) improves survival in a mouse model of polyposis. Kaplan-Meier survival curves where TAM administration was used to induce *Apc* mutation-dependent colon polyposis in *CDX2P-CreER^T2^ Apc^fl/fl^* mice and then mouse cohorts were treated with vehicle (dotted) or RAP (solid) and monitored for survival. Log-rank test comparing the groups treated with RAP or vehicle revealed a significant difference in survival (*p* = 0.03, N = 6 mice per group).

We induced colon polyp formation in *CDX2P-CreER^T2^ Apc^fl/fl^* mice by administration of TAM to activate the function of the CreER^T2^ protein, so that the *Apc* alleles with loxP sites could be somatically mutated by Cre-mediated excision of *Apc* exon 14 in colon epithelial cells. Mice receiving TAM began forming macroscopic polyps within two-three weeks. Polyps progressed rapidly, with regions of dense polyposis apparent in the colon by three-four weeks. Mice were euthanized when near moribund or at defined time points for tissue studies. Mice were treated with 3 mg/kg RAP, which was given for 2 weeks and then stopped for 2 weeks and cycled on and off. The RAP dose and schedule used here was chosen to minimize the adverse effects of RAP. These effects included weight loss and diarrhea in preliminary studies. Treatment with RAP of the mice that had TAM-induced somatic *Apc* inactivation in colon epithelium significantly enhanced survival when compared to treatment with vehicle, p = 0.03, N = 6 mice per group (Figure 1). The median survival of mice receiving vehicle alone was 6.5 weeks versus 21.5 weeks for mice receiving RAP. We measured the body weight in the mice twice per week. We compared mice that were euthanized due to illness (excluding from the analysis those mice euthanized at defined time points), and we did not find significant differences in weight loss between the *CDX2P-CreER^T2^ Apc^fl/fl^* mice receiving vehicle or RAP (18 percent vs 25 percent weight loss) at the time of death. All of the mice that experienced significant weight loss had polyposis. In contrast, the mice in the RAP treatment group that were euthanized after 20 weeks at the completion of the study, rather than due to illness, did not experience weight loss and did not have polyposis. All of the mice that experienced weight loss had polyposis. However, the mice in the RAP group that were euthanized after 20 weeks due to completion of the study, rather than due to illness, did not experience weight loss and did not have many polyps, suggesting that the RAP treatment decreased the weight loss by reducing the polyp formation. We did not witness rectal bleeding in the mice but did note loose stools in both groups. The control mice that did not have Cre recombinase did not experience weight loss with or without RAP.

### RAP Treatment Inhibits Polyp Formation in the Mouse Polyposis Model

**Figure 2:**
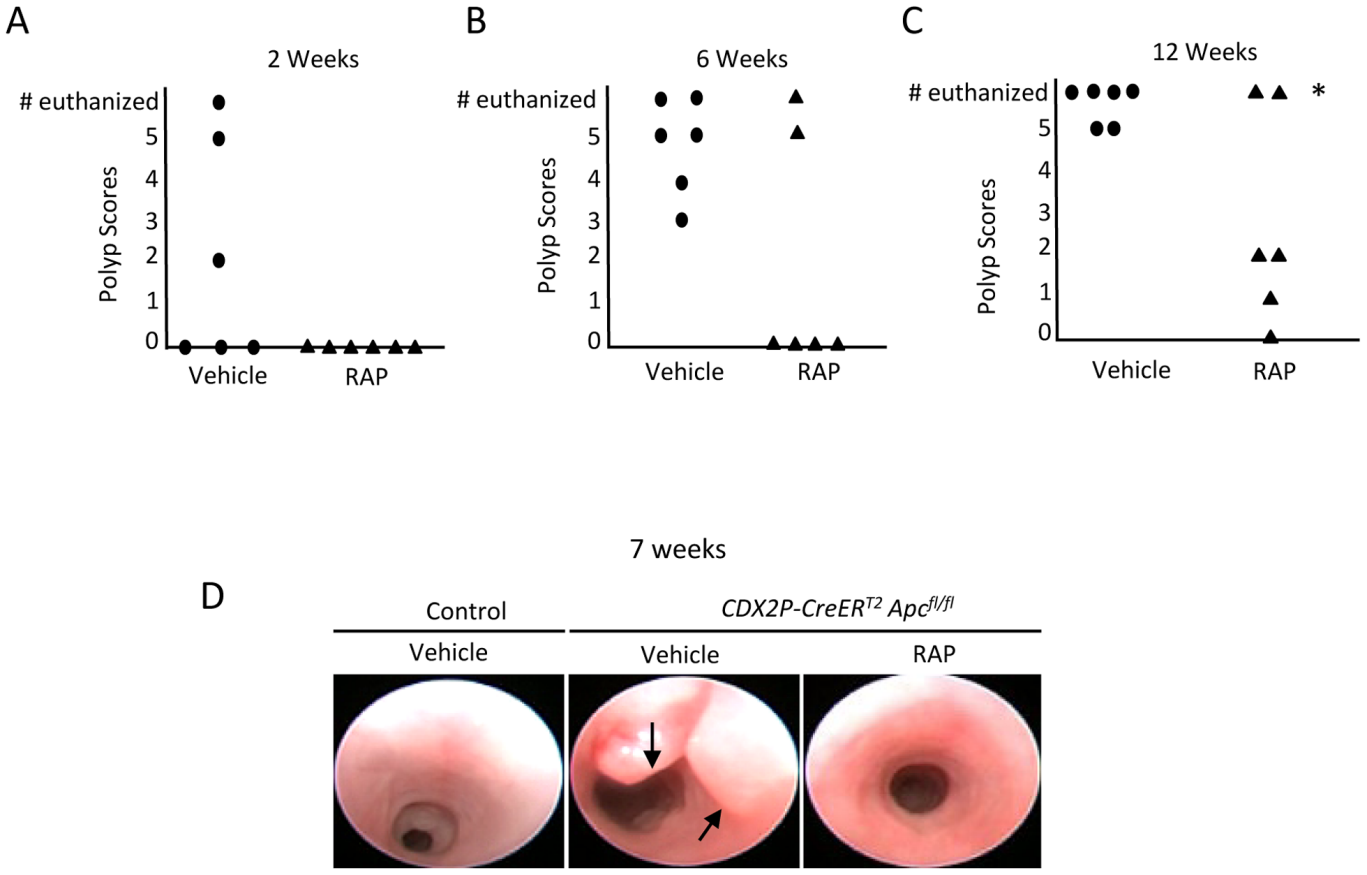
RAP inhibits polyp formation and progression in a mouse model of colon polyposis. (A–C) *CDX2P-CreER^T2^ Apc^fl/fl^* mice were administered TAM to induce *Apc* mutation-dependent polyposis and were then monitored by video-endoscopy for polyp formation after treatment with vehicle (circle) or RAP (triangle). Scores of polyp severity in RAP- and vehicle-treated mice groups were determined at 2, 6, and 12 weeks for all mice that were alive. Each circle or triangle represents an individual mouse (N = 6 per group). The numbers of mice requiring euthanasia due to illness are represented at the top of each graph. The polyp scores were statistically lower by 12 weeks of treatment using 2-sample Z-tests of the proportions of mice with score 4 or higher (N = 6 per group, at 2 weeks, p = 0.12; at 6 weeks, p = 0.08; at 12 weeks, p = 0.01, * signifies p = 0.01). (D) Representative endoscopic pictures of the mouse colons from a control wild-type mouse and TAM-treated and TAM- plus RAP-treated *CDX2P-CreER^T2^ Apc^fl/fl^* mice, with inhibition of polyposis by RAP treatment in the model. Polyps are indicated by black arrows.

We performed endoscopies on each mouse every 1–2 weeks and recorded videos of the endoscopies which were later used to score the colon polyps in a blinded fashion. The cohort of mice that developed polyps following TAM administration and that were treated with RAP had lower polyp scores than the vehicle-treated polyposis cohort at every time point (Figure 2A–C). The polyp scores were statistically lower by 12 weeks of RAP treatment using 2-sample Z-tests of the proportions of mice with score 4 or higher (N = 6 per group; at 2 weeks, p = 0.12; at 6 weeks, p = 0.08; at 12 weeks, p = 0.01).

**Figure 3:**
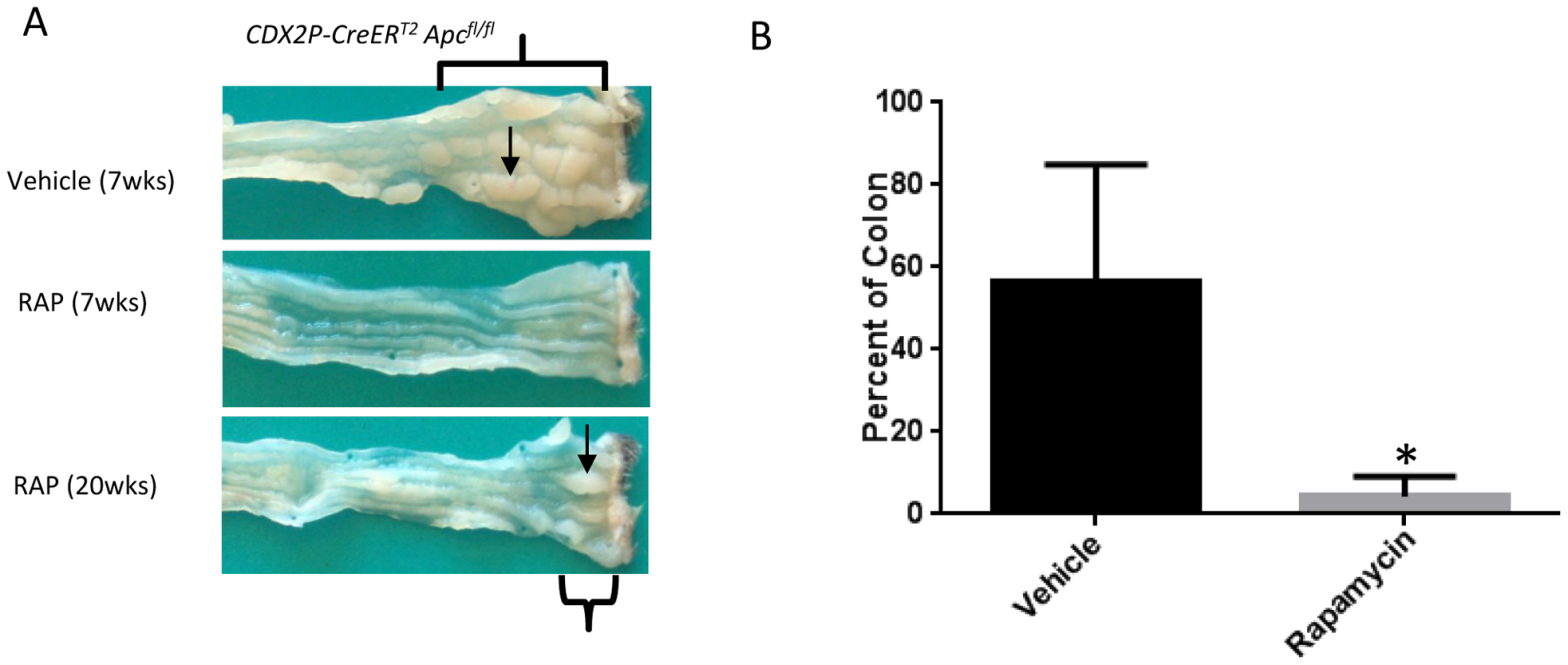
Percentage of the colon with dense polyps is decreased in RAP-treated mice. A.) Gross appearance of colon tissue in *CDX2P-CreER^T2^ Apc^fl/fl^* mice with TAM-induced *Apc* mutation-dependent polyposis plus vehicle treatment for 7 weeks or RAP treatment for 7 and 20 weeks. Note distal colorectal mucosal thickening and visible polyps in tissue from mouse with TAM-induced polyposis treated only with vehicle. Individual polyps are indicated by black arrows. The areas of most dense polyps are indicated by brackets. B.) Mice were monitored and euthanized when ill. The percentage of the distal colon covered with dense polyps at the time of death was found to be significantly lower in the mice receiving RAP (4.3 +/− 2 vs 56.5 +/− 10.8 percent, *p = 0.001, N = 6).

Figure 2D shows representative endoscopic images from a control wild-type mouse and the *CDX2P-CreER^T2^ Apc^fl/fl^* mice, including the absence of colon polyps in a *CDX2P-CreER^T2^ Apc^fl/fl^* mouse with TAM-induced polyposis receiving RAP, as well as the presence of multiple polyps in a mouse in *CDX2P-CreER^T2^ Apc^fl/fl^* mice received only vehicle treatment. Selected RAP and vehicle-treated mice were euthanized at 7 weeks, and photomicrographs of the gross pathology of the large bowel were obtained. Inactivation of *Apc* alleles in colon and rectal epithelium via TAM administration to *CDX2P-CreER^T2^ Apc^fl/fl^* mice led to polyposis in the distal colon, and RAP treatment potently inhibited and delayed polyposis (Figure 3A). A group of mice was monitored and given TAM plus vehicle or RAP and euthanized when ill. The percentage of the colon covered with dense polyps at the time of death was found to be significantly lower in the mice receiving RAP (Figure 3B, 4.3 +/− 2 vs 56.5 +/− 10.8 percent, p = 0.001, N = 6).

**Figure 4:**
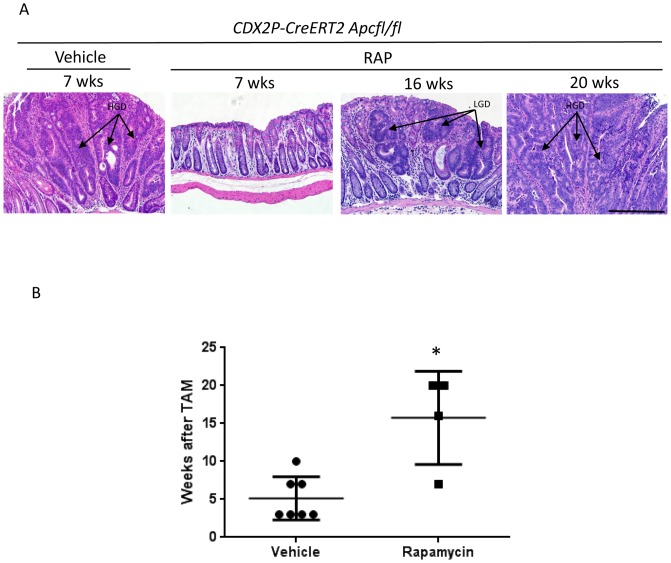
RAP administration delays development of dysplasia in *CDX2P-CreER^T2^ Apc^fl/fl^* mice. *CDX2P-CreER^T2^ Apc^fl/fl^* mice were treated with TAM to induce polyposis and then treated with either vehicle or RAP. A.) Histology of vehicle and RAP treated mice. A group of mice were euthanized at 7, 16 and 20 weeks to compare histology. Arrows indicate areas of low and high grade dysplasia (LGD and HGD, respectively). B.) The second group of mice were allowed to continue until they became ill or beyond 20 weeks of treatment and then were euthanized. Mean number of weeks to visible high grade dysplasia in colonic polyps after TAM induction were recorded for individual mice treated with vehicle (circle) or RAP (square). We found that HGD was seen significantly earlier in the vehicle treated mice vs the RAP treated mice (5.1 vs 15.8 weeks, p = 0.003, N = 7 and 4 in vehicle and RAP group respectively).

### High Grade Dysplasia Identified Later in RAP Treated Mice

A gastrointestinal (GI) pathologist examined representative sections from 8 mice receiving vehicle and 7 mice receiving RAP treatments. Of the mice in the vehicle group, one was euthanized for a 7 week time point and the rest were euthanized because they were moribund with weight loss. Of the mice in the RAP group, 3 mice were euthanized because they were moribund with weight loss, one was a 7 week time point, and 3 were not ill but had lived beyond 20 weeks of treatment.

When the polyps at the time of death were assessed by a GI pathologist, 7 of the 8 vehicle treated colons were found to contain multiple regions of high grade dysplasia (HGD), and 4 of 7 mice receiving RAP had HGD (p = 0.16). Representative images are in Figure 4A. All mice that were euthanized due to weight loss and illness had HGD at the time of death. We then analyzed just the mice where HGD was found and compared the weeks of treatment between the vehicle and RAP treated mice. We found that HGD was seen significantly earlier in the vehicle treated mice vs the RAP treated mice (5.1 vs 15.8 weeks, p = 0.003, N = 7 and 4 in vehicle and RAP group respectively) (Figure 4B).

### RAP Treatment Alters mTOR Signaling, Proliferation and Differentiation Markers, and Expression of β-catenin and Sox9

**Figure 5:**
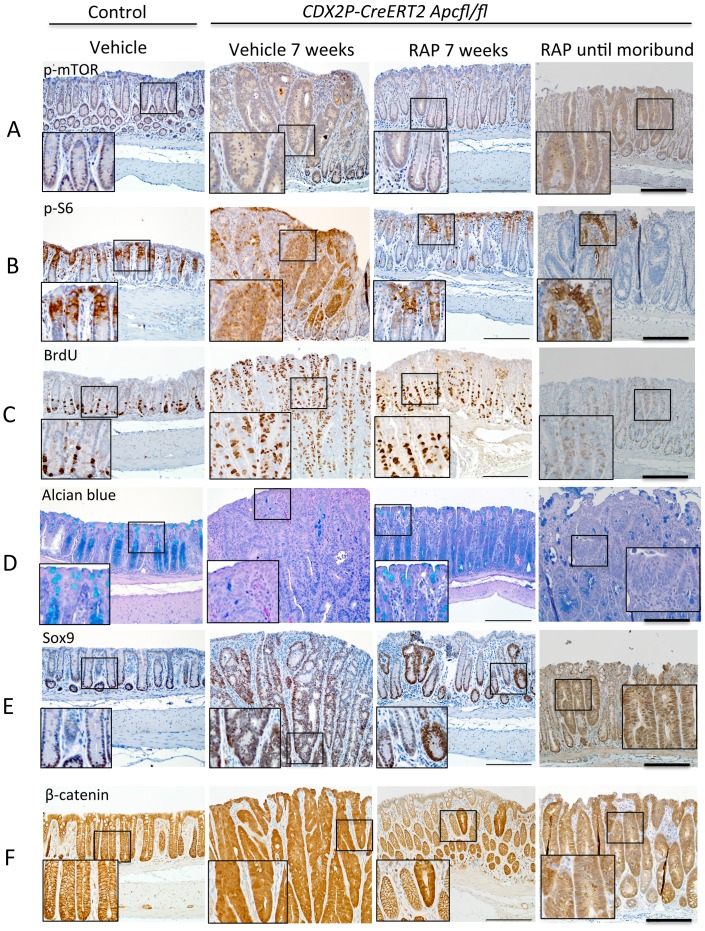
Long-term intermittent dosing with RAP suppresses markers of proliferation and increases differentiation after 7 weeks of treatment (N = 5). (A) and (B) Immunostaining of mouse distal colon shows increased phospho-mTOR (p-mTOR) and phospho-S6 (p-S6) expression in mouse polyps from *CDX2P-CreER^T2^ Apc^fl/fl^* mice injected with TAM. Both increases are suppressed by RAP treatment. (C) BrdU immunostaining showed increased incorporation of BrdU in mouse polyps and RAP partially reversed the effect. (D) Alcian blue staining shows loss of most of the differentiated goblet cells in mouse polyps and RAP restores the goblet cell differentiation. (E) Immunostaining shows increased expression of Sox9 (a β-catenin-regulated cell fate marker) expression in polyps and RAP decreased the expression. (F) Immunostaining shows increased nuclear and cytoplastic β-catenin staining in polyps and RAP treatment restores the membrane staining in most of the cells except a few strongly staining crypts. Mice assessed in the RAP group when moribund had rare areas of polyps with HGD that generally stained like mice in the vehicle group for all markers assessed (column 4, A–F). Scale bars  =  100 µm. The inserts are at two times higher magnification than the original.

RAP has been shown to inhibit mTOR function, and the ribosomal S6 protein is among the proteins known to be phosphorylated by mTOR. When compared at 7 weeks after *Apc* inactivation (N = 5 mice per group), our immunohistochemistry studies revealed increased phospho-mTOR and phospho-S6 staining throughout the *Apc*-mutant adenomatous crypts in TAM-administered *CDX2P-CreER^T2^ Apc^fl/fl^* mice that were treated with vehicle. In comparison, we observed overall modest levels and more focal staining for phospho-S6 and phospho-mTOR in colon tissues of control mice or in colon tissues of *CDX2P-CreER^T2^ Apc^fl/fl^* mice with TAM-induced polyposis that had been treated with RAP (Figure 5A-B). Significantly increased bromo-deoxyuridine (BrdU) incorporation, reflecting increased cell proliferation, was noted throughout the TAM-induced, *Apc*-mutant adenomatous crypts of *CDX2P-CreER^T2^ Apc^fl/fl^* mice that were vehicle treated as compared to RAP treated mice(70.6+/− 1.5 vs 34.2+/−3.4 percent BRDU positive cells; p<0.0001). In addition RAP treatment significantly limited cell proliferation to the lower half of the crypt, similar to the proliferation patterns seen in control colon crypts (Figure 4C). We divided each crypt into 3 equal parts: top, middle, and bottom, and counted BrdU-positive cells and calculated the percent of positive cells in each area in vehicle- and RAP-treated mice at 7 weeks after *Apc* inactivation. We found that the top and middle regions had significantly more positive cells for vehicle-treated vs RAP-treated mice but the bottom of the crypt was not significantly different between the groups (top: vehicle: 74.7+/−2.1 vs RAP: 6.8 +/− 3.1 percent with p<0.0001; middle: vehicle: 70.0 +/−2.5 vs RAP 21.0 +/−5.3 percent with p< 0.0001; bottom: vehicle: 63.4 +/− 2.2 vs RAP 57.8 +/− 3.5 percent with p = 0.1689).

Intestinal mucin, present in differentiated goblet cells, is detected by Alcian blue staining. In TAM-induced, *Apc*-mutant adenomatous crypts of *CDX2P-CreER^T2^ Apc^fl/fl^* mice, we found that Alcian blue staining was largely absent, and RAP treatment restored some of the differentiated goblet cells in *Apc*-mutant adenomatous crypts, albeit not entirely to the numbers seen in control colon epithelium (N = 5) (Figure 5D). The percentage of Alcian blue-positive cells was significantly higher in the RAP-treated group at 7 weeks after TAM injection (vehicle: 1.6 +/−0.3 vs RAP: 86.6 +/−1.2 percent, P<0.0001).

Increased cytoplasmic and nuclear staining for β-catenin and increased nuclear staining for the β-catenin-regulated Sox9 transcription factor were seen throughout the TAM-induced, *Apc*-mutant adenomatous crypts of vehicle-treated *CDX2P-CreER^T2^ Apc^fl/fl^* mice (N = 5) (Figure 5E and 5F). In contrast, in control colon crypts, β-catenin staining was largely confined to the membrane and Sox9 staining was only seen in cells at the crypt base (Figure 5E, 5F). RAP treatment of *CDX2P-CreER^T2^ Apc^fl/fl^* mice largely restored β-catenin and Sox9 staining to the patterns seen in control mice, with the exception of a few strongly staining crypts (N = 5) (Figure 5E, 5F). Neither lysozyme staining for Paneth cell differentiation nor TUNEL staining for apoptotic cells were significantly different when vehicle- and RAP-treated groups of TAM-administered *CDX2P-CreER^T2^ Apc^fl/fl^* mice were compared (data not shown). The polyps with dysplasia at the time of death in the mice receiving RAP who became moribund were assessed histologically and by immunohistochemistry and were found to be similar to those in the mice receiving vehicle in staining patterns (fourth column, Figure 5). The staining in these polyps was not quantified as these areas of dysplasia were rare (n = 2–3). Additionally, as seen in Figure 2A–C, the polyps took longer to be visible on endoscopy.

**Figure 6:**
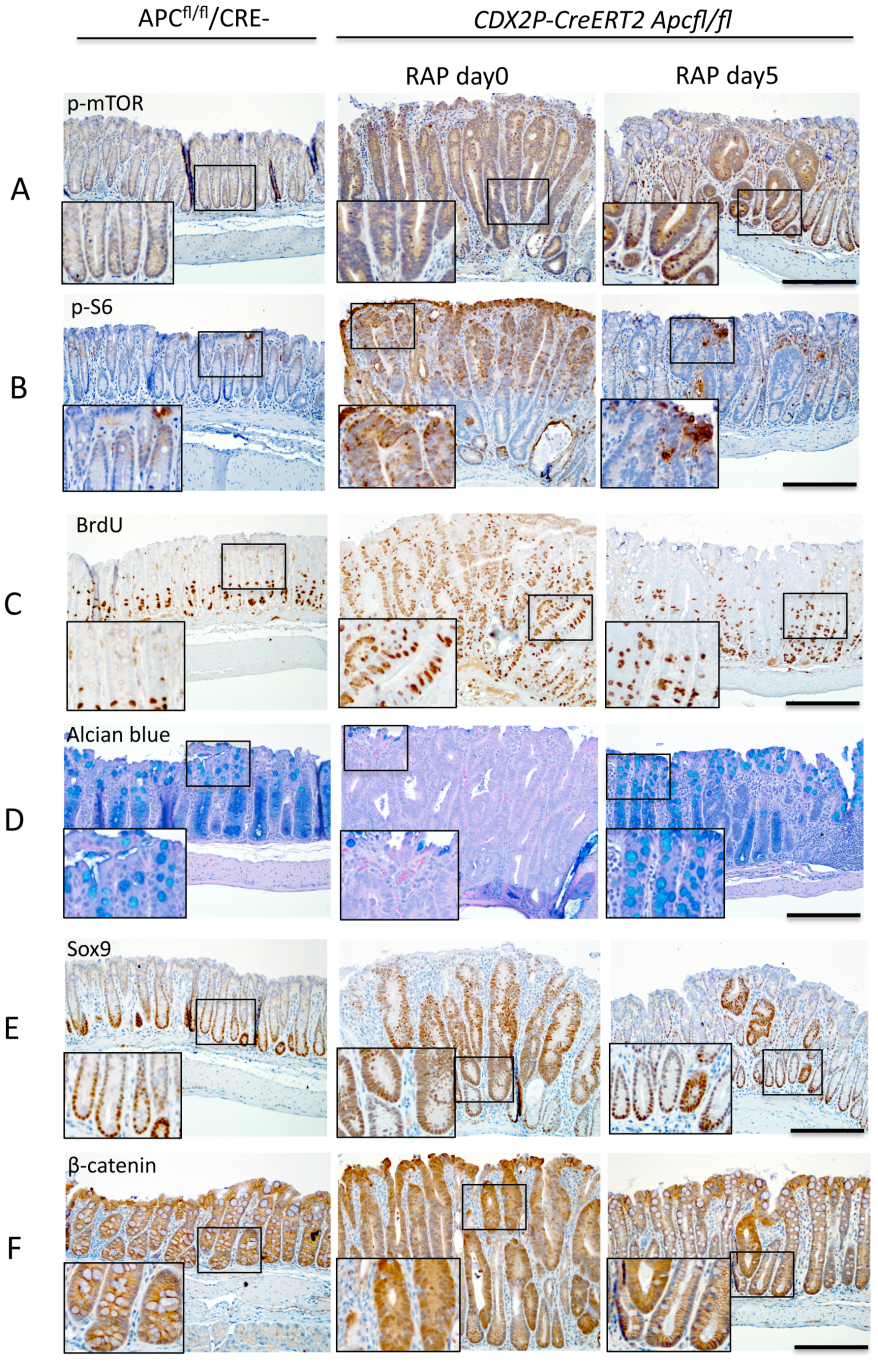
A brief course of RAP treatment decreases markers of proliferation and increases markers of differentiation. *CDX2P-CreER^T2^ Apc^fl/fl^* mice were injected with TAM and monitored until polyposis was seen endoscopically, then the mice were treated with vehicle or RAP at 3 mg/kg concentration for periods of one, three, or five days (N = 5 per timepoint). (A) and (B) Immunostaining of distal colons from these mice or Cre negative *Apc^fl/fl^* mice for phospho-mTOR and phospho-S6. Both phospho-mTOR and phospho-S6 show increased expression in mouse polyps due to *Apc* inactivation, which is suppressed by 5 days of RAP treatment. (C) Immunostaining of BrdU showed increased incorporation of BrdU in mouse polyps and RAP partially reversed the effect. (D) Alcian blue staining shows loss of most of the differentiated goblet cells in mouse polyps and RAP restore the goblet cell differentiation. (E) Immunostaining shows increased Sox9 (a cell fate marker) expression in polyps and RAP decreased the number of cells expressing Sox9. (F) Immunostaining shows increased nuclear and cytoplastic expression of β-catenin in polyps and RAP treatment for 5 days decreased the expression. Scale bars = 100 µm. The inserts are at two times higher magnification than the original.

To address the potential means by which RAP might be acting to inhibit polyposis, in a second group of mice (N = 5 per time point), we inactivated *Apc* in the colon epithelium of *CDX2P-CreER^T2^ Apc^fl/fl^* mice by TAM administration and followed the mice endoscopically until they had dense large polyps. The colon epithelium of the mice was then examined via immunohistochemical approaches after polyposis was induced (no RAP treatment) and after the mice had been treated with RAP for periods of one, three, or five days. The mice receiving RAP were found to have decreased staining for phospho-mTOR and phospho-S6 in colon crypts, along with decreased incorporation of BrdU and increased Alcian blue staining, particularly so by day 5 of RAP treatment (Figure 6A–D). Staining for days 1 and 3 were intermediate. We quantified the percent BrdU-positive cells in the mice that had developed polyposis and that had been treated with vehicle or RAP for 5 days, and we found that the colon epithelium of the vehicle-treated mice had significantly higher percent BrdU-positive cells as compared to the RAP-treated group (86.1 +/−1.2 vs 38.3 +/−2.0, p<0.0001) (number of mice  = 5). We divided each crypt into top, middle and bottom compartments and we found significantly greater mean percent BrdU-positive cells in each compartment (top: 76.2 +/− 2.9 vs 5.1 +/− 1.5 percent for vehicle vs. RAP, p<0.0001; middle: 84.8 +/− 2.4 vs 13.8 +/−3.2 percent for vehicle vs. RAP, p< 0.0001; bottom: 85.5 +/− 1.8 vs 54.8 +/− 2.5 percent for vehicle vs. RAP, p = 0.0001). In addition, we quantified the percent of alcian blue-positive cells per crypt and found that after 5 days of RAP, there were significantly more alcian blue-positive cells in RAP-treated mice (vehicle: 1.9 +/−0.6 vs RAP: 86.8 +/−3.0 percent, P<0.0001). Lysozyme and TUNEL staining showed no differences between vehicle- and RAP-treated groups (data did not shown).

### RAP Does Not Antagonize the β-catenin-dependent Wnt Signaling Pathway in Intestinal Epithelial Cells

**Figure 7:**
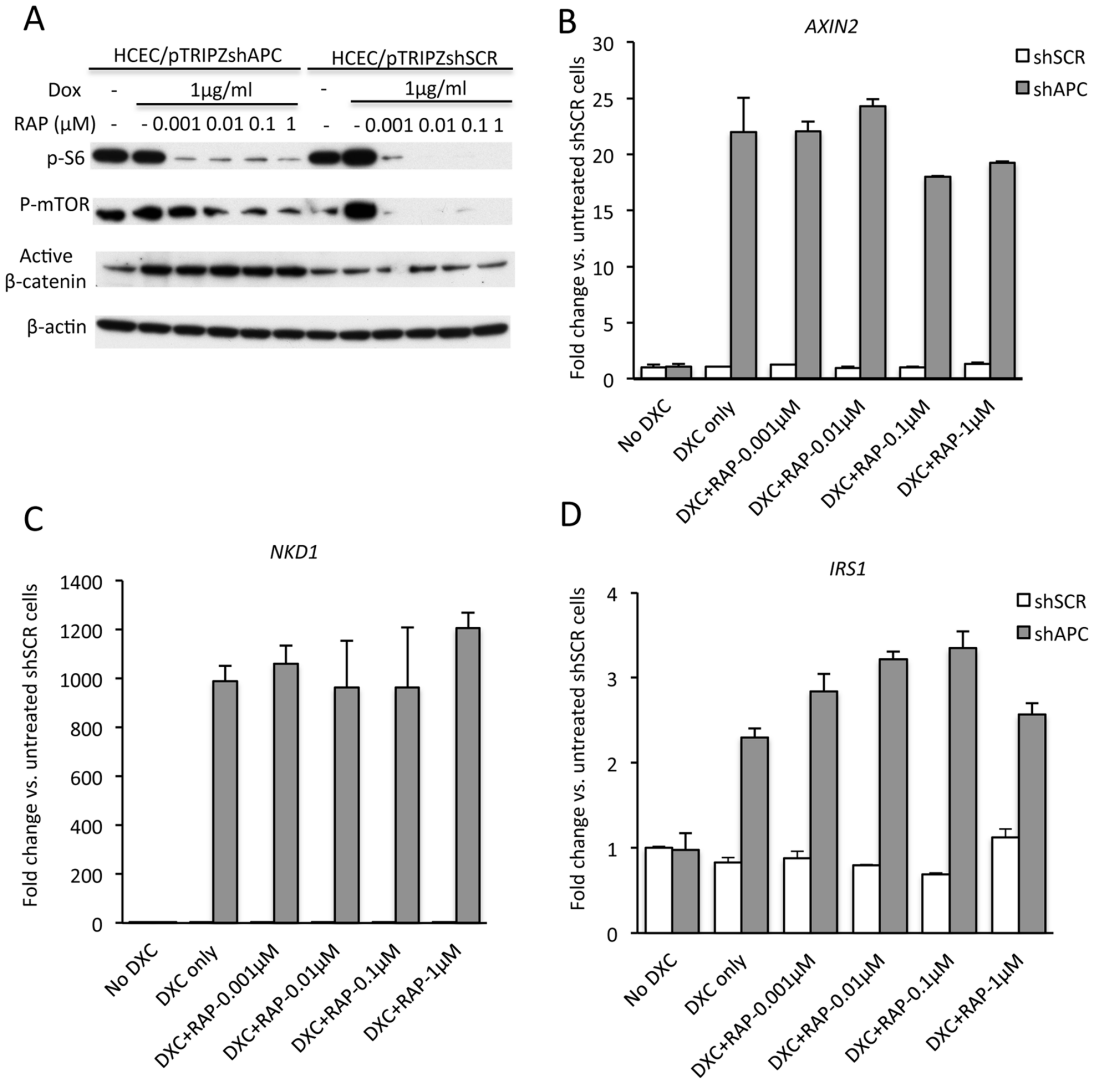
RAP inhibits mTOR signaling in HCECs in a β-catenin/TCF transcription-independent manner in vitro. HCEC lines stably transformed with shAPC (HCEC/ptripzshAPC) or shScramble (HCEC/ptripzshSCR) were treated with Doxycycline (DXC) at 1 µg/ml to induce shRNA expression for 48 hr or untreated (-). The cells were then treated with RAP (0.001–1 µM) or vehicle (−) for 24 hr in the presence of Doxycycline under serum starving condition (0.2% serum). Total cell lysates were analyzed by Western blotting for phospho-S6 and phospho-mTOR. β-actin protein levels served as loading control (A). Significant decrease in phospho-S6 and phospho-mTOR levels was observed in cells treated with RAP. Expression of selected β-catenin/TCF-regulated target genes *AXIN2*, *NKD1* and *IRS1* were analyzed in the same cells by real time PCR (B–D). The results represent the mean ± SEM; n = 5 for each treatment group. There were no statistical differences between groups.

To further address the possible means by which RAP acts to inhibit Apc mutation-dependent colon polyp formation and progression, we studied immortalized human colon epithelial cells (HCECs) where APC expression was markedly inhibited via shRNA-mediated approaches. HCECs stably expressing a shRNA targeting APC or a control scrambled shRNA were treated with vehicle or various concentrations of RAP. Western blot showed that phospho-mTOR and phospho-S6 were significantly decreased by RAP in a dose-dependent manner but that active β-catenin [11], which is presumably responsible for Wnt signaling, is not decreased (Figure 7A). Using real-time PCR, we found that the expression of downstream transcriptional targets of the β-catenin/TCF signaling pathway that are known to be elevated in colorectal adenoma and carcinomas, such as *AXIN2*, *NKD1*, and *IRS1*, were not decreased by RAP treatment in the HCECs (Figure 7B–7D). These findings suggest that the inhibitory effects of RAP on polyposis occur via mechanisms independent of direct effects on the β-catenin/TCF-dependent signaling pathway.

## Discussion

The studies and data here demonstrate the utility of RAP in polyp prevention in a mouse colonic model of FAP. In this model, mice are given an inducing agent and both copies of *Apc* are inactivated in many colon crypts, leading to severe polyposis. We found significantly improved survival, decreased polyp formation, and prolonged time to development of dysplasia with RAP treatment. We found changes linked to increased proliferative features, such as increased β-catenin and Sox9 expression and increased BrdU labeling in polyps, and these changes were countered by short or long term dosing with RAP. We also saw decreased Alcian blue staining, indicating a loss of differentiated goblet cells, and the decrease in goblet cells was markedly reversed by as little as 5 days of RAP treatment. To further investigate the relationship between the mTOR pathway and the Wnt signaling pathway, we pursued focused studies of the effects of RAP on β-catenin/TCF-regulated target genes in cultured, immortalized HCECs where endogenous *APC* expression was dramatically inhibited by shRNA-based approaches. We did not find evidence that RAP directly affected the expression of β-catenin/TCF-regulated genes in the cultured HCECs, and the means by which RAP dramatically inhibits *Apc*-mutation dependent polyposis in mouse colon epithelium *in vivo* is likely independent of direct effects on the β-catenin-dependent Wnt signaling pathway.

Prior cell culture-based studies of RAP in colon cancer-derived cell lines have offered mixed results. Nozawa and co-workers treated eight different colon cancer cell lines with various concentrations of RAP and did not find any significant change in proliferation [4]. However, Shao and colleagues did find RAP inhibited proliferation in an intestinal epithelial cell line with an inducible expression construct expressing a K-Ras^Val12^ mutant protein [12]. Yet another study from Ponnurangam and colleagues reported that Tandutinib, which inhibits mTOR signalling, decreased proliferation in SW480 cells and in HCT116 xenografted tumors growing in immunocompromised mice, but the effect of Tandutinib was not purely through the Akt/mTOR pathway [13].

Prior studies in Apc^min/+^ mice, where polyp formation is predominantly in the small intestine and animals die of anemia and weight loss, showed that adding RAP to their food decreased polyp formation, improved anemia, decreased weight loss, and improved the defects in colon electrolyte transport [14]. In this study by Koehl, the authors acknowledged that they did not see an effect on colon polyps, which are rare in Apc^min/+^ mice. Our mouse model produces extensive polyposis in the distal colon and our findings demonstrate substantive effects of RAP in inhibiting colon polyposis and the development of high grade dysplasia in colon epithelium. As such, our findings indicate that quite dramatic inhibitory effects of RAP on Apc mutation-dependent tumorigenesis are also clearly evident in colon epithelium.

Prior animal studies have found that Everolimus, another mTOR inhibitor, decreased polyp formation in the small intestine and reduced mortality in APC^Δ716^ mice [2]. The mechanism for the in vivo effects of Everolimus on intestinal polyp formation in the APC^Δ716^ mice was not elucidated. The authors of that work did in fact provide data indicating that siRNA mediated inhibition of β-catenin expression in the SW480 and DLD-1 colorectal cancer cell lines decreased both total and phosphorylated mTOR levels. Studies and data on the possible effects of mTOR inhibition on downstream β-catenin/TCF-regulated transcriptional targets were not addressed. While endoscopic evaluation of the regression of individual *Apc*-mutant mouse colon polyps following RAP dosing has been described [15], [16], the studies in this paper are to the best of our knowledge the first to show dramatic prevention of polyposis formation and/or progression in an *Apc* mutation-dependent colonic polyposis model. The advantages of the model of colon polyposis used in the studies here relative to previously described mouse models include the following: I) dramatic colon polyposis is induced in adult mice at a defined time point by TAM administration; II) a relatively predictable pattern and number of colon polyps arise within a three-four week period; and III) individual mice can be serially studied by endoscopy to assess changes in polyposis over time, including the effects of potential inhibitors of polyposis development and/or progression.

There are currently no definitive medical management alternatives to proctocolectomy for the long-term clinical management of FAP patients. Because proctocolectomy can have potentially significant complications and life-altering effects, improved medical management strategies to forestall surgery would be highly desirable. RAP is unlikely to be a strong candidate for in-depth clinical studies in FAP patients, due to its side effects such as immune suppression and inhibition of healing [17], [18]. Other clinically useful inhibitors of mTOR, with less significant side effects than RAP, may have greater potential promise. We used RAP for the studies here as a proof-of-principal reagent for mouse colon polyposis inhibition due in large part to cost consideration issues. Another potential use for drugs inhibiting mTOR in FAP patients might be to prevent upper gastrointestinal tract polyps, especially as pancreaticoduodenectomy, which is necessary when upper tract polyposis becomes too extensive to manage endoscopically, is associated with considerable morbidity.

Further studies that better discern the mechanism of inhibition of colon polyposis by mTOR inhibitors in mouse models are expected to be of considerable benefit in developing new approaches and agents for the inhibition of polyposis in FAP patients and perhaps in other patient populations with APC-mutant colorectal tumors.
